# Increasing Optical Path Lengths in Micro-Fluidic Devices Using a Multi-Pass Cell

**DOI:** 10.3390/mi15070820

**Published:** 2024-06-25

**Authors:** Victor Argueta-Diaz, McKenna Owens, Ahmed Al Ramadan

**Affiliations:** 1 Department of Physics and Engineering, Alma College, Alma, MI 48801, USA; 2 College of Engineering, University of Michigan, Ann Arbor, MI 48109, USA; owensmck@umich.edu; 3 Al Qahtani PCK Pipe Company, Dammam 31462, Saudi Arabia; ahmed.rr95@gmail.com

**Keywords:** microfluidics, long-optical path, lab-on-a-chip

## Abstract

This study presents a novel absorption cell with a circular geometry that can be integrated into microfluidic devices for optical spectroscopy applications. The absorption cell is made of PDMS/SU8 and offers an optical path length that is 8.5 times its diameter, resulting in a significant increase in the sensitivity of the measurements. Overall, this design provides a reliable and efficient solution for optical spectroscopy in microfluidic systems, enabling the precise detection and analysis of small quantities of analytes.

## 1. Introduction

Lab-on-chip systems (LOCs) represent a shift in the field of analytical chemistry and biomedical engineering, offering opportunities for miniaturization, and integration of complex laboratory processes onto a single microscale device [1,2,3]. The relevance of LOC systems stems from their transformative potential to revolutionize various fields, including healthcare, environmental monitoring, pharmaceuticals, and point-of-care diagnostics. These systems enable rapid and cost-effective analysis of biological and chemical samples with minimal sample volumes, reduced reagent consumption, and enhanced portability compared to traditional laboratory setups.

One of the key driving forces behind the increasing relevance of LOCs is their ability to address the growing demand for personalized and precision medicine [2,4,5]. By enabling the integration of multiple analytical functions onto a single chip, LOC platforms empower healthcare professionals to perform rapid and accurate diagnostic tests at the point of need. This capability is particularly crucial for early disease detection, monitoring of treatment efficacy, and tailoring therapies to individual patients’ needs. Moreover, LOC systems facilitate the development of novel diagnostic tools. In [6,7], the authors present the use of microfluidic chips for single-cell metabolite analysis, showcasing how these chips allow for precise observation, classification, and stimulation of individual cells. The integration with advanced detection equipment provides detailed metabolic profiles at the single-cell level, crucial for understanding cellular heterogeneity and metabolic processes.

Micro-optical fluid devices hold significant relevance in overcoming the challenges posed by the small volumes inherent in microfluidic systems, particularly in the context of optical detection techniques. While optical methods offer non-invasive and non-destructive means of analysis, the limited sample volumes in microfluidic devices present obstacles for techniques like optical spectroscopy, which often require long optical paths to effectively capture small absorption signals. This constraint arises due to the reduced interaction length between light and analytes within the microfluidic channels, leading to diminished sensitivity and accuracy in detection.

To address this challenge, researchers have proposed innovative solutions, among which multi-pass cells have emerged as a promising approach. Traditionally, multi-pass cells such as the White cell and the Herriott cell [8,9] have been utilized to extend the optical path length by facilitating multiple reflections of light within the cell through arrangements of spherical mirrors. Although effective, these configurations require significant space, and careful mirror alignment, and are constrained in terms of size reduction while remaining compatible with the fabrication processes of LOC devices. Therefore, the development of micro-optical fluid devices aims to adapt and optimize multi-pass cell technology for integration into compact microfluidic platforms, enabling enhanced optical detection capabilities while overcoming spatial constraints and ensuring compatibility with microscale systems.

In this article, we introduce a novel circular multi-pass cell designed specifically for optical spectroscopy measurements within microfluidic environments [10,11,12]. Our proposed multi-pass cell, constructed using SU8 and PDMS on a silicon substrate, represents a departure from conventional reflective surface configurations. Instead, it uses the principle of total internal reflection (TIR) to effectively guide light within the microfluidic channel, thereby mitigating associated losses. Our design eliminates the need for intricate mirror arrangements and precise alignment, offering a simplified yet robust solution for enhancing optical detection capabilities in microfluidic systems.

The utilization of SU8 as the fabrication material ensures optimal compatibility with microfluidic devices [13,14,15], facilitating seamless integration and minimizing potential structural complexities. Moreover, the incorporation of PDMS further enhances the versatility and functionality of the multi-pass cell, enabling flexibility in design and accommodating diverse experimental requirements. This approach not only addresses the challenges posed by the limited spatial constraints of microfluidic environments but also underscores the importance of cost-effective and scalable solutions for advancing optical spectroscopy techniques in biomedical and analytical research domains.

This article is organized as follows: Section 2 presents the design justification and explains how to estimate the optical path length in the multi-pass cell. In Section 3, we discuss the fabrication process, modifications to the PDMS curing process, and introduce a prototype fabricated on a silicon wafer. Section 4 describes the experimental setup, compares the absorption lines of ethanol and water using our multi-pass cell and a commercial cuvette, and examines transmission loss. Finally, in Section 5, we provide conclusions and outline potential future work.

## 2. Design

The objective of our investigation was to detect small concentrations of substances such as *E. coli* or pollutants present in water samples. To fulfill this objective, we designed a cell featuring an extended optical path length, thereby facilitating the detection of small concentrations of target substances within a solution. Our design entails a multi-pass circular cell configuration, wherein light travels a ring-shaped pathway multiple times before exiting through the designated output port.

Circular multi-pass cells are innovative optical devices designed to enhance the interaction between light and sample within a confined space. These cells operate on the principle of multiple reflections, wherein a beam of light entering a circular mirror at an angle θ propagates along a star-shaped trajectory. This trajectory, known as a star polygon, is characterized by parameters such as the number of spikes *p*, and the density of the star *q*, which determine the path of the light within the cell [10,11,12,16]. By exploiting total internal reflection and carefully controlling the incidence angle of the incoming beam, circular multi-pass cells can achieve a significantly extended optical path length compared to conventional optical configurations. This prolonged interaction between light and sample enhances the sensitivity and accuracy of spectroscopic measurements.

The incident angle can be calculated as [10]:

(1) $$\theta =\frac{p-2q}{2p}\pi$$

The parameter *q* in circular multi-pass cells denotes the division of the corresponding arc of one side on the circumscribed circle of the regular star polygon pattern into *q* segments by other vertices. It is important to note that both *p* and *q* are relative prime integers, meaning they share no common factors other than 1. This property ensures that the star polygon pattern remains well-defined and allows for efficient control over the trajectory of light within the cell. Additionally, the angle θ is limited to ensure that TIR occurs in the SU8-air interface, which requires that θ to be at least $48^{\circ }$.

**Figure 1:**
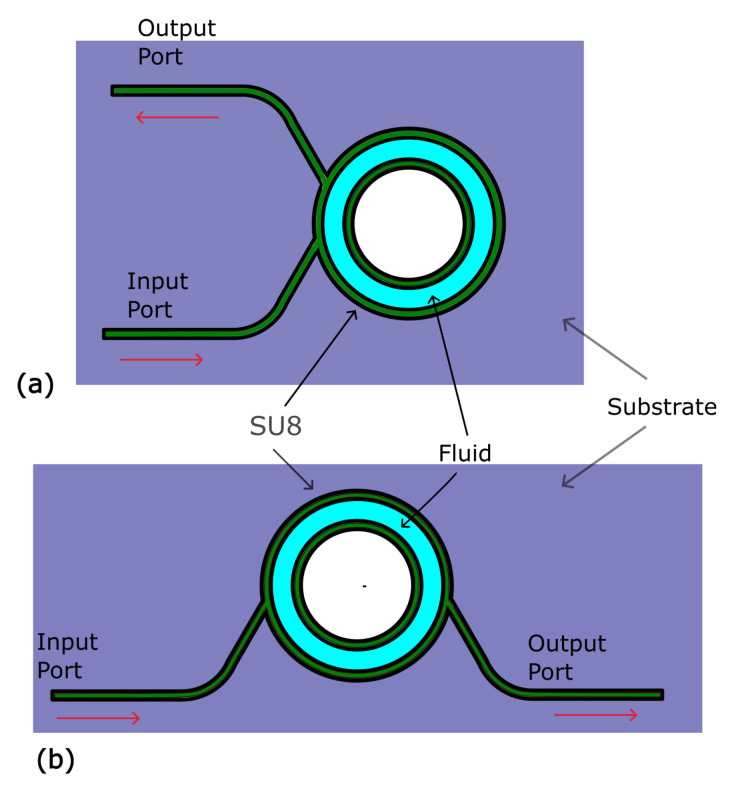
Top view of proposed designs for a circular multi-pass cell. (**a**) both input and output ports are on the same side and their optical path length is defined by Equation (2), in (**b**) input and output ports are on the opposite side allowing for an easier experimental implementation, although the optical path length is reduced by half. The PDMS layer is not shown.

For the initial design, the optical path length can be quantitatively calculated using the formula established by Markus et al. [10]. This equation tailors the design parameters to achieve optimal optical path lengths tailored to specific analytical requirements.

(2) $$l_{opt}=pD\cos\left(\theta \right)$$

where *D* is the cell’s diameter. In the case of the design of Figure 1b, we need to add a 0.5 factor.

As an illustrative example, let us consider a scenario where the release angle is set at 65 degrees, and the diameter of the circular multi-pass cell measures 10 mm. Under these conditions, we can anticipate a total of 20 bounces (p = 20) within the cell. Consequently, the optical path length is projected to extend approximately 84.5 mm, representing a substantial increase of 8.4 times the diameter of the multi-pass cell. This significant enhancement in optical path length underscores the efficacy of the multi-pass cell in facilitating prolonged light-sample interactions within a compact spatial footprint.

Furthermore, it is noteworthy that diverse optical path lengths can be achieved by manipulating key parameters such as the diameter of the inner ring and the release angle. By reducing or even eliminating the diameter of the inner ring and adjusting the release angle to increase the number of bounces before reaching the output port, researchers can tailor the optical path length to suit specific analytical requirements.

**Figure 2:**
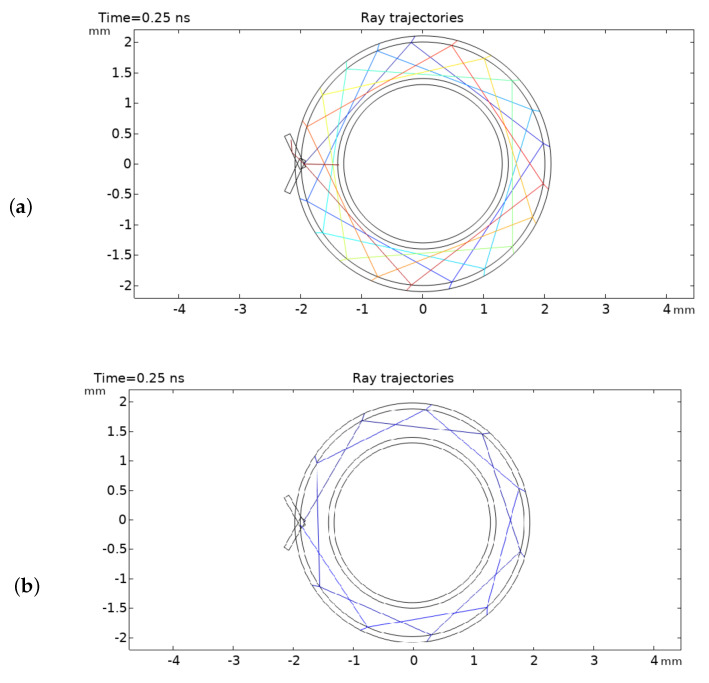
Ray optic simulation. The optical path can be adjusted by changing the release angle and size of the inner ring. (**a**) Shows an OPL of 8.4× with a release angle of 65 degrees. (**b**) Shows an OPL of 11× with a release angle of 48 degrees.

For example, by modifying the release angle to 48 degrees while concurrently reducing the diameter of the inner ring, the optical path length can be further extended to approximately 11 times the diameter of the outer ring. This versatility in achieving variable optical path lengths underscores the adaptability and utility of the circular multi-pass cell for diverse spectroscopic applications within microfluidic platforms. For instance, Figure 2 depicts two optical ray simulations wherein an optical path length equivalent to 8.4 times and 11 times the diameter is achieved.

Figure 1 shows a top view of our proposed designs. In Figure 1a, both input and output ports are on the same side and their optical path length is defined by Equation (2), in Figure 1b, the input and output ports are on the opposite side allowing for easier experimental implementation, the trade-off of an easier implementation, however, is that the optical path length is reduced by half. In this article, we use the second design as a proof of concept of our system.

The structural composition depicted in Figure 1 comprises three discernible components: an inner ring and an outer ring, both fashioned from SU8 material (depicted in green) and a centrally positioned hollow cavity designated for fluid circulation (illustrated in blue). Additionally, the configuration encompasses a substrate, which may be crafted from glass, a silicon wafer, or PDMS, and a PDMS cover. To elaborate, the central cavity facilitates fluid introduction through the PDMS cover (not shown), while the input optical guide facilitates the insertion of light into the cavity. It is pertinent to emphasize our expectation of primarily working with fluids possessing a refractive index lower than that of SU8 (*n* = 1.57).

Light entering the outer ring undergoes reflection back into the central cavity via total internal reflection (TIR) between SU8 and air on the outer ring. This mechanism ensures that the light path within the circular multi-pass cell is elongated, thereby enhancing the interaction between light and the fluid medium. The utilization of total internal reflection (TIR) within the cell confers a remarkable advantage, enabling the realization of a long optical path length within the confined dimensions of a mere couple of square centimeters on a microfluidic chip. This innovative mechanism not only maximizes the utilization of limited space but also amplifies the interaction between light and the sample, thereby augmenting the sensitivity and efficacy of optical spectroscopy measurements.

## 3. Fabrication

The design depicted in Figure 1b has been selected for fabrication due to its advantageous configuration, which features input and output ports on opposing sides of the structure. This arrangement facilitates a convenient experimental setup. We used a modified soft lithography process to fabricate these designs combining PDMS, silicon, and SU-8 layers.

PDMS, renowned for its simplicity in fabrication and cost-effectiveness, emerges as the material of choice for molding within numerous microfluidic devices [17]. Its inherent flexibility and compatibility with a wide range of experimental conditions render PDMS an ideal candidate for constructing the foundational framework of our circular multi-pass cell. Meanwhile, SU-8 assumes the role of the waveguide material, owing to its exceptional optical properties and seamless transferability of designs through lithographic processes [13]. Through this integration of PDMS and SU-8, we aim to realize a robust and functional circular multi-pass cell capable of delivering reliable and high-performance optical spectroscopy measurements within microfluidic environments.

### 3.1. SU-8 Development

As stated previously, we will use soft lithography to create our system. To begin this process, we first clean a standard silicon wafer with three acetone and DI water rinses, finishing with a bake at ${200\;}^{\circ }$C for 10 min to ensure the wafer is completely clean of any impurities. Once the wafer had cooled upon removal from the hotplate, we then used a high-frequency generator to remove any last impurity that could interfere with this fabrication.

With the cleaned and prepped silicon wafer in place, the spin-coating step ensues, marking the initiation of the layer deposition process. A droplet of OmniCoat, a specialized solution serving as a binder for SU-8, is meticulously dispensed onto the wafer surface, ensuring comprehensive coverage. The spin-coating operation is meticulously executed, involving sequential spins at 500 rpm for 9 s followed by 1300 rpm for 49 s. These specific rotational speeds are meticulously selected to achieve an optimal and uniform layer of OmniCoat across the wafer surface. After the spin-coating step, the wafer undergoes another brief bake at ${200\;}^{\circ }$C for 1 min to solidify the deposited layer.

Upon cooling, the fabrication process progresses with the application of SU8-100, the primary material for constructing the desired structure. Similar to the OmniCoat application, the SU8-100 solution is evenly dispersed across the wafer surface, followed by a series of spin-coating cycles at 500 rpm for 9 s and 2000 rpm for 49 s. This meticulous spin-coating procedure is critical in ensuring precise control over the thickness and uniformity of the SU8 layer. Subsequently, the wafer, now laden with the SU8 layer, undergoes a thermal treatment regimen, involving exposure to elevated temperatures of ${65\;}^{\circ }$C for 20 min followed by ${95\;}^{\circ }$C for 70 min. This thermal curing process is indispensable for solidifying the SU8 layer and establishing the desired structural integrity essential for subsequent processing steps.

Following the preparatory steps, the SU-8 layer is now primed for exposure to the designated design pattern. Employing masks created from black-and-white photographic film, the desired design is precisely positioned atop the SU-8 layer. This mask serves as the blueprint for transferring the intricate features onto the SU-8 substrate. The assembly, comprising the mask and SU-8 layer, is subsequently subjected to controlled UV exposure. To ensure optimal exposure the assembly is irradiated for a duration of 4 s, resulting in a cumulative energy dosage of approximately 500 mJ/cm^2^. This carefully calibrated exposure time and power level strike a delicate balance, facilitating the accurate transfer of the design onto the SU-8 layer while minimizing the risk of detrimental overexposure.

Upon completion of the UV exposure step, the mask is carefully removed, unveiling the transferred design pattern on the SU-8 layer. The post-exposure processing continues with a thermal treatment regimen, involving a brief post-exposure bake at ${65\;}^{\circ }$C for 1 min followed by a more extensive curing period at ${95\;}^{\circ }$C for 12 min. This thermal treatment step is essential for consolidating the exposed SU-8 regions and ensuring the structural integrity of the fabricated design.

Subsequently, the fabricated slide undergoes a rinsing process using the SU-8 developer solution, a critical step in removing the unexposed SU-8 regions and revealing the final design. Careful attention is paid to the duration of rinsing, with a timeframe of 1 min established to prevent over-corrosion or underdevelopment of the design. Intermediate rinsing intervals with DI water at 30-s intervals ensure thorough removal of developer residue. Following the rinsing process, pressurized nitrogen is employed to meticulously dry the slide, ensuring the absence of any residual moisture or contaminants.

**Figure 3:**
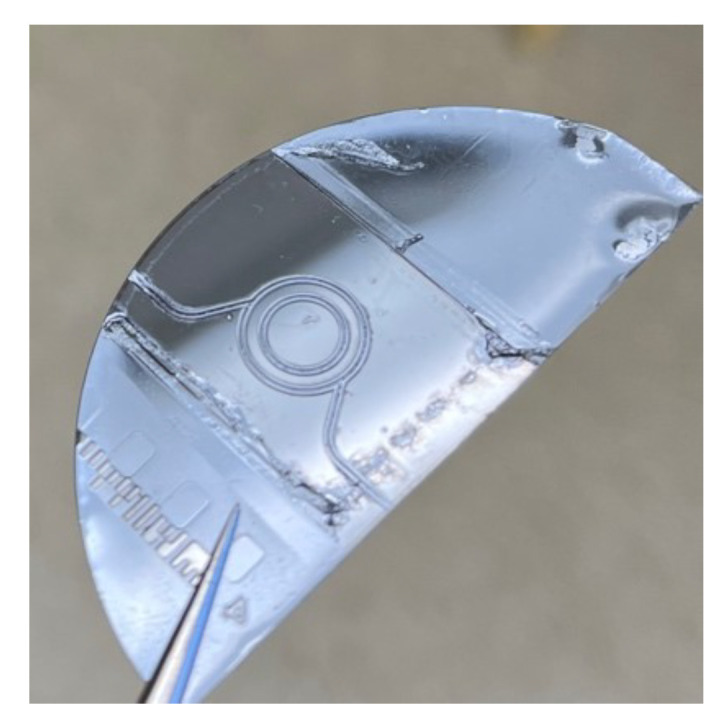
Fabricated multi-pass cell. The figure shows the multi-pass-cell fabricated on a silicon wafer before adding the top PDMS layer.

The final stage of the fabrication process entails a concluding thermal treatment step, involving a brief bake at ${95\;}^{\circ }$C for 5 min. This step serves to halt any further development processes and stabilize the fabricated design, culminating in the realization of the final structure. The completed design is shown in Figure 3.

### 3.2. PDMS Fabrication

After completing the SU-8 layer, the next step involves fabricating a flat PDMS layer to serve as a lid atop the SU-8 structure. This PDMS layer functions as a seal, effectively confining both the solution and the circulating light within the cell. PDMS fabrication entails the utilization of a two-part kit comprising a pre-polymer (base) and a curing agent, as recommended by the manufacturer [17]. Adhering to the specified 10:1 ratio, the pre-polymer and curing agents are mixed to ensure optimal polymerization and material properties.

Following the mixing process, the PDMS mixture undergoes degassing for a duration of 2 h to eliminate any entrapped air bubbles, ensuring uniformity and integrity of the resulting PDMS layer. Subsequently, the degassed PDMS mixture is carefully poured onto a flat surface, where it is uniformly spread to form the desired flat layer. The assembled PDMS layer is then transferred to an oven set at ${65\;}^{\circ }$C, where it undergoes a curing process for a duration of one hour. This controlled thermal treatment is essential for promoting cross-linking reactions within the PDMS matrix, thereby solidifying the material and establishing the desired mechanical properties.

Sometimes, however, it is important to manipulate the refractive index of PDMS, enabling the design and realization of fundamental optical components such as waveguides, beam splitters, or diffuse reflectors. Various techniques are available for controlling the refractive index of PDMS, including adjustments to the curing temperature, or curing using UV light [18,19,20,21]. In this paper, we present in Table 1 the effects of altering the curing temperature on the refractive index for four different base-to-agent ratios. These measurements are conducted prior to bonding the PDMS with the multi-pass cell. However, in previous experiments, we have not observed significant changes in these measurements after the bonding process [22].

**Figure 4:**
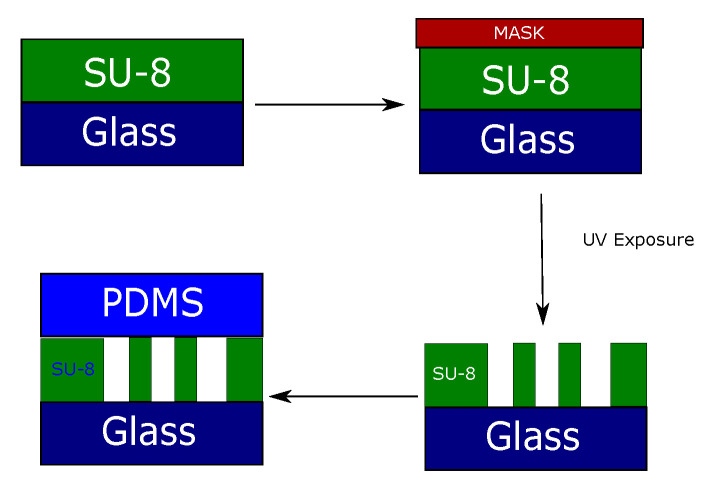
Cell fabrication process. SU8 is used as the waveguide material; the substrate can be glass, PDMS, or silicon.

The fabrication process involving SU-8 and PDMS layers is summarized in Figure 4, providing a visual representation of the sequential steps culminating in the realization of the final microfluidic cell structure.

## 4. Results and Discussion

Following the fabrication process, our attention turned towards the testing phase of our device. To commence testing, we aligned a 1 mW HeNe laser using a 10× microscope objective mounted onto a multi-axis stage, affording the setup five degrees of freedom. This alignment ensured the focused delivery of incoming light into the input port of the fabricated cell, thereby initiating the experimental setup.

Once the laser alignment was successfully accomplished, we proceeded to substitute the laser with a broadband light source. This light source was coupled through the microscope objective, allowing for the transmission of light into our cell. Within the confines of the cell, the transmitted light underwent several reflections before exiting through the output port. The light exiting the output port was directed into a commercial Spectrometer, facilitating the comprehensive analysis of spectral characteristics and enabling the extraction of valuable data pertaining to the optical properties of the cell.

**Figure 5:**
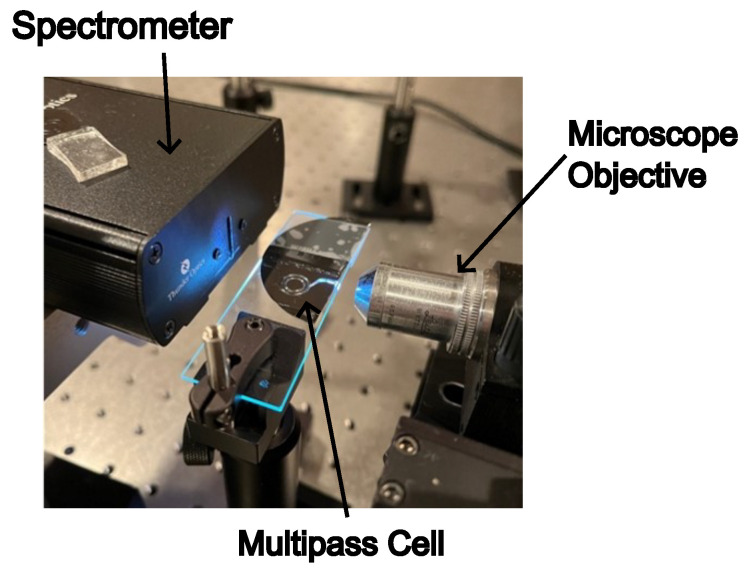
The experimental setup. The figure shows a light source illuminating a microscope objective, the multi-pass cell on a translation stage, and a spectrometer.

This experimental setup is visually depicted in Figure 5, showing the integration of the multi-axis stage, our fabricated cell, and the spectrometer. Through this testing setup, we aimed to validate the functionality and performance of our device.

To evaluate the performance of our circular cavity cell, we initiated a comparative analysis with a spectrometer system utilizing a quartz cuvette. The assessment involved recording spectra from the commercial spectrometer using a cuvette containing solutions of distilled water and ethanol at varying concentrations. Subsequently, we replicated these measurements using our circular cavity cell under identical experimental conditions.

We initiated the testing process by recording spectra from the spectrometer when only air was present between the PDMS layer and the cell. Subsequently, we introduced a water–ethanol concentration of 5% into the cell to examine the spectral differences. This was achieved by carefully depositing a drop of either solution onto the cell using a 1 mL syringe, followed by covering it with our PDMS layer. After each deposition, spectra were recorded, and the PDMS layer along with the solution was removed from the cell. This process was repeated several times for each solution to ensure consistency and reliability of the results.

**Figure 6:**
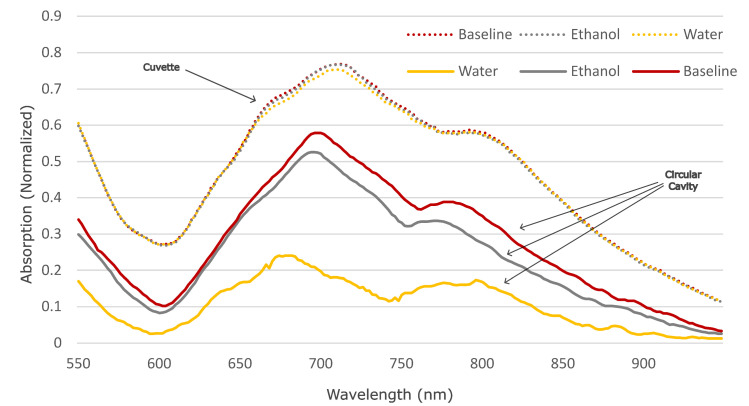
The figure illustrates a comparison of the absorption characteristics of alcohol and water when measured using our multi-pass device and a cuvette. The data from the cuvette measurements are represented by dotted lines, providing a reference for comparison. The y-axis of the graph shows the normalized absorption. Different peaks correspond to absorption from silicon, SU8, PDMS, water, and ethanol.

We were able to discern notable differences in absorption between the varying solutions. These differences are indicative of the sensitivity of our cell to changes in sample composition, thus affirming its efficacy for spectroscopic analysis within microfluidic environments. The conclusive demonstration of our cell’s ability to detect differences in absorption between different solutions is visually depicted in Figure 6, highlighting its potential as a versatile and reliable tool for a wide range of analytical applications.

During the experimental procedure, both the commercial spectrometer system and our circular cavity cell were subjected to the same samples of distilled water and ethanol solutions at different concentrations. Spectral data obtained from each measurement were meticulously analyzed and compared to discern any disparities or similarities in performance between the two systems.

By conducting this comparative analysis, we aimed to ascertain the efficacy and reliability of our circular cavity cell in facilitating spectroscopic measurements in microfluidic environments. We sought to gain valuable insights into the performance characteristics and potential advantages offered by our fabricated cell in comparison to conventional spectrometer systems utilizing quartz cuvettes.

The spectral data obtained from our circular cavity cell underscored its efficacy in discerning differences in absorption across the tested solutions, thereby validating its utility as a robust tool for spectroscopic analysis within microfluidic environments. These findings are illustrated in Figure 6. The peaks at 600, 675, and 750 correspond to the absorbance created from the light interaction with the silicon wafer, ethanol, PDMS, SU8, and water [23,24,25,26]. For more significant measurements it would be necessary to move to an IR band, where the absorption of water increases significantly [23,24].

Upon careful examination of our experimental results, a distinct pattern emerges: the employment of the multi-pass cell consistently yields higher sensitivity in detection compared to traditional methods. This enhanced sensitivity can be attributed to the increase in the optical path length created by the multi-pass cell design. As light travels through the multi-pass cell, it undergoes multiple reflections and interactions with the sample (and surrounding material, leading to a prolonged and intensified interaction. This extended interaction duration ultimately amplifies the sensitivity of the detection system, enabling the detection of even subtle variations in sample composition.

In contrast, when conducting identical measurements using a traditional cuvette, significant limitations become apparent. The obtained results exhibit reduced visibility and discernibility within the given scale, posing challenges in the accurate interpretation of spectral data. This limitation underscores the inherent constraints associated with employing a conventional cuvette for spectroscopic measurements.

Looking ahead, we would like to expand the band range to include the far-infrared region to better enhance the capabilities of our optical sensor. Working in the infrared spectrum, as opposed to the visible spectrum, offers several distinct advantages. Firstly, many molecular vibrations and rotations that are critical for the identification and analysis of various substances occur in the infrared range [27]. This makes the infrared spectrum particularly suitable for detailed chemical and biological sensing applications.

Secondly, infrared light has better penetration capabilities through certain materials that are opaque in the visible spectrum, such as biological tissues [28]. This characteristic is invaluable for medical diagnostics and non-invasive monitoring, allowing for deeper tissue imaging and more accurate assessments.

Furthermore, infrared sensors are less susceptible to interference from ambient light, which improves the signal-to-noise ratio and enhances the precision and reliability of measurements [29]. This is particularly important in environments with variable lighting conditions, where maintaining consistent and accurate sensor performance can be challenging.

### Losses

Losses in micro-optical devices are crucial considerations due to their direct impact on device performance and functionality. These losses can occur due to various factors such as absorption, scattering, and reflection within the optical components. In micro-optical devices, where size constraints are paramount, minimizing losses is essential to ensure efficient light transmission and accurate signal detection.

Losses directly affect the signal-to-noise ratio (SNR) of the device. High losses result in a weaker signal relative to background noise, making it challenging to discern the desired signal from noise. This can significantly degrade the sensitivity and reliability of the device, limiting its ability to detect and analyze target analytes accurately. Losses, also, contribute to reduced optical power reaching the detection or measurement components of the device. This diminishes the overall efficiency of the device, necessitating higher input power levels to achieve desired output signals.

**Figure 7:**
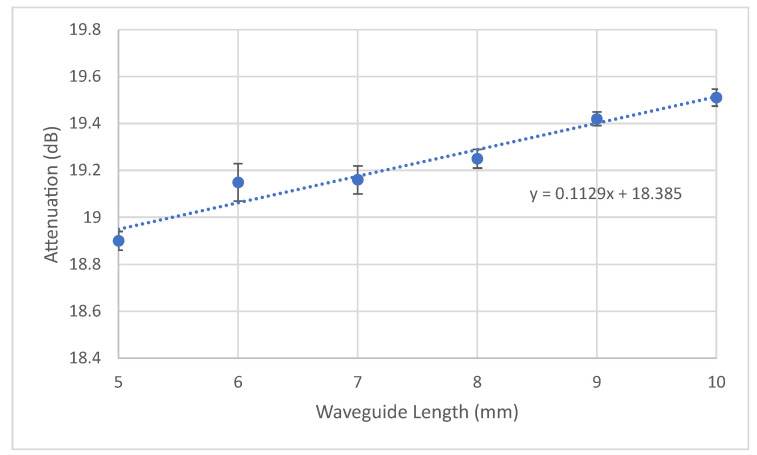
Transmission loss at 650 nm. Trend line calculated using linear least square. average STDEV of ±0.047 dB.

In our study, we conducted an assessment of the attenuation characteristics of the proposed design. This involved systematically reducing the length of the waveguide from 10 mm to 5 mm across six incremental steps. The resulting attenuation data, illustrated in Figure 7, reveals a calculated attenuation loss of 1.2 dB/cm ± 0.047 dB/cm, with an insertion loss of around 18 dB, which is in the range of what has been previously reported [30].

This attenuation value is significant as it indicates the decrease in light intensity over the length of the waveguide. However, it is important to note that even with this level of attenuation, the optical power remains sufficient for various applications, particularly in the context of compact biosensors where space is limited. Therefore, this attenuation level is deemed acceptable and still enables the effective utilization of the proposed design in lab-on-chip systems and other related applications.

## 5. Conclusions

We have successfully developed a compact yet highly effective multi-pass circular cell for optical spectroscopy, occupying only a small area of a few square centimeters. We have achieved an optical path length approximately 8 times the diameter of the cell, crucial for enhancing sensitivity and precision in spectroscopic measurements. This achievement is made possible by leveraging the principle of total internal reflection (TIR) between the walls of our innovative design, ensuring prolonged light-sample interactions within the confined space of the cell.

Utilizing PDMS and SU-8 materials in the construction of our multi-pass circular cell offers several distinct advantages. Not only does this combination facilitate ease of reproduction and modification, but it also enables seamless integration into existing microfluidic platforms. Furthermore, our new design exhibits significantly higher sensitivity compared to traditional cuvettes, while analyzing a smaller volume of the solution. This enhanced sensitivity, coupled with the ability to analyze smaller sample volumes, positions our device as a valuable tool for a wide range of spectroscopic applications.

## Figures and Tables

**Table 1:** Refractive index of PDMS at different curing temperatures and times.

Ratio/Temperature	50 Celsius (60 min)	50 Celsius (120 min)	100 Celsius (60 min)
2.5:1	1.414	1.416	1.427
5:1	1.4135	1.415	1.423
10:1	1.413	1.4146	1.419
20:1	1.412	1.4133	1.4175

## Data Availability

The original contributions presented in the study are included in the article, further inquiries can be directed to the corresponding author.
